# Parental resources and heritability as factors shaping children's health. An analysis of twins' self-rated health using TwinLife

**DOI:** 10.3389/fsoc.2023.1136896

**Published:** 2023-06-27

**Authors:** Bärbel Holzwarth, Christof Wolf

**Affiliations:** ^1^School of Social Sciences, University of Mannheim, Mannheim, Germany; ^2^GESIS Leibniz-Institute for the Social Sciences, Mannheim, Germany

**Keywords:** parental resources, children's health, gene-environment interaction, ACE-variance decomposition, heritability

## Abstract

We assess the relative and joint contributions of genetic and environmental factors on health during childhood and assume that parental resources are part of the environmental factors shaping children's health. We discuss theoretical background and empirical evidence concerning the effects of parental resources and heritability on children's health. Based on these findings we formulate six hypotheses guiding our empirical analysis, using data from TwinLife, a nationally representative sample of same sex twin pairs in Germany. We analyze self-rated health of 1,584 twin pairs aged 4–18. We did find strong support for the idea that parental resources influence children's health: household income and fathers' education consistently show positive effects. In contrast to our expectation, we did not find that genetic factors influence the health of well-off children less than the health of children living in families with lower SES. We also did not find that the genetic influence on health increases during childhood and adolescence. On the contrary our results indicate that the role played by genetic factors diminishes whereas environmental factors gain importance for health of children while growing up. This finding is good news for those interested in improving health chances of children from lower SES backgrounds because it demonstrates the malleability of children's health.

## 1. Introduction

Health is an important condition that enables individuals to participate in every area of life. Understanding health differences between individuals is therefore important. We investigate two central factors influencing health: resources and heritability. As both factors can influence differences in health, we are interested in their relative contributions to health of children.

Individuals with less resources are generally at higher risk to experience worse health than individuals with more resources (Syme and Berkman, 1976; Bartley, 2004; Lampert, 2016). Because we are interested in children's health, but children still depend on their parents' resources, we study resources comprising the socioeconomic position of households; that is education of parents, household income, and parental employment status. Social groups with similar resources are assumed to have comparable living situations and life experiences, and thus, similar behavior and opportunities (Ditton and Maaz, 2011, p. 193–194). But health is not only influenced by resources but also by heritability, also referred to as genetics or genetic factors.

A common approach to assess heritability is the use of twin studies which make use of genetic information provided by the zygosity of the twins: monozygotic (MZ) twins share 100 percent of their genes and dizygotic (DZ) twins—like non-twin sibling pairs-−50 percent of their genes (e.g., Schur et al., 2009; Hatemi et al., 2010). This allows twin studies to differentiate between genetic and environmental factors. For the purpose of this study and in line with the definition of “environment” in quantitative genetics (Plomin et al., 2013, p. 95–96), environmental factors are all factors other than genetic ones, not limited to but including parental resources. With the twin method, as we will see later, “environment” can further be decomposed in environmental conditions shared by the twins and those unique to each twin. To genetic factors we also refer to as heritability.

We focus on children and adolescents aged 4–18 years. Studying children's health is essential for at least the following two reasons: First, children are a special social group because inter alia their health depends on parental resources, as children have no or only little resources of their own (Mayall, 1998). Second, childhood health affects adult life chances (Bartley, 2004; Dragano and Siegrist, 2009; Dietscher and Pelikan, 2016). Health issues occurring in childhood can trigger chronic illness and limit the quality of life in adulthood (Lampert and Schenk, 2004, p. 58). Therefore, disentangling the contribution of parental resources and genetic factors on child health can inform policies aiming to reduce health inequalities for children.

We study the effect of parental resources and heritability on self-rated health of children. Asking for self-evaluation of one's health is a common way to assess health because it covers not only the objective but also the subjective perception of health (Poethko-Müller et al., 2018). Previous research shows that self-rated health is associated with physical and mental health and that it can predict morbidity, not only for adults but also for children and adolescents (Vingilis et al., 2002; Breidablik et al., 2008; RKI, 2008). Self-rated health was also shown to be a good, in the USA perhaps even an increasingly better predictor of mortality (Schnittker and Bacak, 2014).

Recent research on genetic effects on health differences claims that genetic factors can explain 32–63 percent of variance in self-rated health of adults (for instance, Røysamb et al., 2004; Silventoinen et al., 2007). However, no study has so far analyzed the genetic and environmental contribution to *children's* self-rated health. In contrast, a broad body of research shows that parental resources affect children's self-rated health (for instance, Case et al., 2002; Chen et al., 2002; Currie and Stabile, 2002; Huurre et al., 2003; Reinhold and Jürges, 2012; Nakamura, 2014). Other studies consider both, genetic factors and resources when analyzing self-rated health of adults aged 46–69 years (Osler et al., 2007). Yet, no study has analyzed the relative contribution of parental resources and heritability to self-rated health simultaneously. We address this research gap by decomposing the variance in children's self-rated health into heritability and parental resources.

Our research question is: *How much of the variance in child health can be explained by parental resources and how much by genetic factors?* Since childhood is a phase of enormous development, we are further interested in the dynamic of parental resources and genetic factors on self-rated health, more specifically, how the contributions of both factors to health change during childhood. Moreover, we are interested in how resources moderate the influence of genetic factors.

This article is structured as follows: We focus on the theoretical background of heritability and the association of parental resources and children's health in combination with giving an overview of the current research on twin studies on health as well as parental resources and health. After deriving our hypotheses, we introduce TwinLife, the data set we use in our analysis as well as the chosen analytical strategy. Next, we present the results and conclude with a critical discussion of results and implications for future research.

## 2. Theoretical background and previous research

In Germany, more than half of the children (57%) aged 0–17 years report very good self-rated health, almost 40 percent report good health and only around 4 percent below good health (Kuntz et al., 2018). Although variance in health decreased during the last years (Lampert et al., 2019), health differences remain. These differences in children's health are shaped by genetic and environmental factors. To disentangle causes for variance in health further, we discuss three broad fields of theoretical background and research in the following. Firstly, we briefly discuss the role of heritability and parental resources. Secondly, we assume that parental resources are, among other factors, part of the environmental factors shaping children's health. Lastly, we focus on the interaction of heritability and environmental factors. After the discussion of our theoretical considerations we present pertinent research. We focus on national and international studies analyzing self-rated health of children using representative samples with twins and non-twins.

### 2.1. Heritability

Children receive half of their mother's and father's genes randomly (Plomin et al., 2013, p. 34). The trait outcome of interest in our research, i.e., the phenotype, is child health. Heritability is defined as the proportion of observed variance among individuals caused by genetic differences (Plomin et al., 2013, p. 86–87). If heritability of a phenotype is high, genetic factors mostly explain differences between individuals. In quantitative genetics, variance that genetic differences cannot explain is ascribed to the environment. Studies of genetic and environmental impacts—or nature vs. nurture—aims to assess their relative contribution to observed differences in phenotypes (Rijsdijk and Sham, 2002). This relative contribution can vary between different groups or traits. Whenever changes in environmental or genetic influences happen, the relative contribution of environment and genetics is altered (Plomin et al., 2013). Hence, the role of genetics and environment for differences in a phenotype are context-dependent and vary with, for example, age, sex, or country (see section on gene-environment interactions below).

Heritability is commonly assessed with twin studies. These studies capitalize on the fact that monozygotic (MZ) and dizygotic (DZ) twins are distinguished by the amount of genes they share. Like non-twin siblings, DZ twins are 50 percent genetically alike, whereas MZ twins share 100 percent of their genes. A higher variance in an outcome for DZ twins compared to MZ twins indicates genetic impact (Schur et al., 2009). The variance that genetic factors cannot explain is per definition caused by environmental factors. These are further distinguished into *common* environment—environmental factors that increase the similarity within pairs of twins, e.g., family home—and *unique* environment—environmental factors that differ within pairs of twins, e.g., circle of friends, but also random variation and measurement error (Plomin et al., 2013; Harden, 2021).^1^

Our assumption that genetic factors affect children's health is confirmed by empirical evidence. We discuss twin studies that disentangle the contributions of genetic and environmental factors to self-rated health. To our knowledge there are no twin studies for children studying environmental and genetic factors in relation to self-rated health. Therefore, we rely on results from adolescent and adult twins [for instance, age 18–31 years (Røysamb et al., 2004), age 16–25 (Silventoinen et al., 2007), age 26–86 (Svedberg et al., 2005), age 17–91 (Svedberg et al., 2001)]. Among these studies, the proportion of genetic, common and unique environmental factors differs considerably when explaining variance in health (Røysamb et al., 2004; Silventoinen et al., 2007).

Røysamb et al. (2004) disentangle the contribution of environmental and genetic factors on health by analyzing 6,576 twin pairs aged 18–31 years of a cross-sectional Norwegian twin study. Common environmental factors explain 32–37 percent, unique environmental factors 30–35 percent and genetic factors 28–38 percent of the variance in self-rated health, with slight differences of results for men and women (Røysamb et al., 2004). We will discuss current research on heritability in more detail in the section after next. In this context, we also discuss a twin study that operationalizes health with BMI instead of self-rated health (Johnson et al., 2019) to learn particularly about children.

### 2.2. Parental resources

Material living conditions are among the major determinants of health. In a systematic review Moor et al. (2017) conclude that material factors have a stronger effect on self-rated health than psychosocial or behavioral factors, a finding that holds across age or gender groups. Material living conditions of children are shaped by their parents' resources. The association of parental resources and child health is well established (for instance, Case et al., 2002; Currie and Stabile, 2002; Huurre et al., 2003; Doyle et al., 2007; Reinhold and Jürges, 2012; Bøe et al., 2014; Nakamura, 2014; Jones, 2018). Financial means and education reflect the family's position in the social hierarchy and shape children's health. Parental income determines the possibility to invest in child health. This can be through services or activities such as a membership in a sports club or traveling but also goods such as healthy diet or good housing quality. Likewise, parental income affects what children can afford. Both aspects can decide about inclusion or exclusion of children from joining everyday activities which in turn also affects health (Levitas et al., 2007; Lampert and Richter, 2009).

During childhood, children and their health depend on parental education. On the one hand, education is a commodity. Educational degrees help individuals to get jobs. Jobs create income, and thereby, influence what individuals can afford (Ross and Mirowsky, 2010). On the other hand, education “enables people to coalesce health-producing behaviors into a coherent lifestyle” (Mirowsky and Ross, 2003, p. 25) and thereby increases personal sense of control over one's health both of which foster health of parents and children. In other words, education affects health above and beyond the effect it has on occupational prestige and income.

Differentiating between different types of parental resources makes it easier to think about pathways through which children's health is affected. For example, mother's education may be an indicator for her interest in and knowledge of child nutrition, while household income likely will be related with quality and type of housing. Both factors can affect children's health but through very different pathways. However, as Link and Phelan (1995) have forcefully argued, social inequalities are a *fundamental* cause of health. Thus, by focusing on particular pathways one runs the risk of losing sight of inequality as such. They emphasize that it is impossible to trace all possible pathways by which social inequalities are linked to health and that when one of these pathways is blocked new ones arise. In order to incorporate this view into our analysis we also analyze the effect of the socioeconomic status (SES) of the household on children's health.

Research on the relationship of parental resources and children's health is broad and well established (for instance, Black et al., 1992, p. 118–122; Case et al., 2002; Currie and Stabile, 2002; Huurre et al., 2003; Doyle et al., 2007). In line with our theoretical argument that money and knowledge are the most important resources affecting health, we discuss nationally representative studies that focus on the association of parental resources, measured as parental income or education, and children's health, operationalized as self-rated health. We also discuss two studies that operationalize health other than subjectively measured (e.g., mental health, body weight) as these studies disentangle the role of parental resources further by analyzing parental resources separately for mother and father. Finally, we discuss three studies analyzing the health effects of the socioeconomic status of parents on children's health.

Reinhold and Jürges (2012) investigate the effect of parental income on child health in a German sample of children aged 0–17 years. The results support the income gradient in parent-rated child health, that is, children's health increases with increasing parental income. This effect remains even when controlling for parental education (Reinhold and Jürges, 2012). A study on a Japanese sample of children aged 7–15 years supports the results by Reinhold and Jürges (2012). Again, parental income and children's self-rated health are positively associated (Nakamura, 2014). In a cross-sectional analysis of the Bergen Child study, Bøe et al. (2012) investigate the effect of education and income on mental health of children aged 11–13 years. According to this study children with higher family income and better parental education have better mental health (Bøe et al., 2012). Interestingly, when considering the effect of parental income on child's weight in a longitudinal U.S. sample, parental income is not significantly predicting child's weight whereas education, especially father's education, plays an important role (Jones, 2018).

Some of these studies indicate that the association of parental resources and child health can differ by parent. Bøe et al. (2012) find that whereas lower education of both, mother and father, is associated with higher conduct problems, hyperactivity and inattention, only lower education of fathers is associated with higher emotional problems (Bøe et al., 2012). Similarly, Jones (2018) finds that particularly fathers' education predicts children's weight: with increasing education of the father, the child's risk for becoming overweight decreases. Jones (2018) further considers parental employment status, arguing that depending on their employment status, parents might have less time to care for their children's nutrition, for example, by preparing healthy meals. In fact, the more weeks per year a mother is working the higher is the probability for a child to be overweight. While father's employment is also positively associated with child's weight this result is not statistically significant. Nakamura (2014) analyzes employment status finding support for the results reported by Jones (2018). Until the age of 11, mothers' employment seems to be positively related to children's obesity. In contrast, fathers' employment seems to be negatively associated with children's obesity, however, this association is not significant.

Instead of analyzing the effects of single resources on health some studies use an index of different resources featuring as socioeconomic status and analyze its effects on children's health. Based on a longitudinal study on the health of children, adolescents and young adults in Germany, Kuntz et al. (2018) investigate cross-sectionally how children's health differs by socioeconomic status (SES; *N* = 15,023; children aged 0–17) where SES is composed of parental education, occupation and income. Results show a social gradient in self-rated health with children with lower SES experience worse health (Kuntz et al., 2018). This finding is confirmed by Rattay et al. (2022) who analyze the same dataset focusing on adolescents and their parents (*N* = 3,556; adolescents aged 11–17). Not only do they find a relationship of income (only for girls), education and occupational status separately with self-rated health but also of an SES index and health. Another German study investigating cross-sectional data of the LIFE Child study (children aged 3–18) also finds a social gradient in child health, operationalized with BMI: higher SES-scores (also composed of parental education, occupation and income) are associated with lower BMI (Poulain et al., 2019).

### 2.3. Gene-environment interaction

Genetics and environment can contribute independently to variance in a phenotype, but it is also possible that both factors interact with each other: depending on the environment the effect of genetics on the phenotype can differ. Similarly, depending on the combination of genes, environmental effects on the phenotype can differ (Purcell, 2002; Plomin et al., 2013). This means that depending on the environment, heritability of a phenotype can be higher or lower. Accordingly, high heritability does not imply that environmental factors are unimportant; they might suppress or promote the effects of genetic factors (gene-environment interaction). Put differently, genetic effects may not be homogeneous within a population but can differ between individuals depending on their environment (Purcell, 2002).

Current research provides examples on how environmental factors can moderate the contribution of genetic factors. For instance, Johnson et al. (2010) find that good education can serve as a buffer for genetic factors promoting bad health. Among adults with higher education the contribution of genetic factors on variance in self-rated health is smaller (Johnson et al., 2010). This does not mean that individuals with low levels of education are genetically more vulnerable to poor health. The authors assume that it could rather mean that high education can suppress genetic vulnerabilities (Johnson et al., 2010, p. 412).

Similar to the result found by Johnson et al. (2010), another study shows that parental SES can moderate genetic influences on child BMI, especially for girls: With higher parental SES the influence of genetic factors on children's BMI decreases (Johnson et al., 2019).

### 2.4. Dynamics of the influence of parental resources and heritability on child health

In addition to the interaction of parental SES and genetic factors just mentioned, there may also be an interaction of children's age and heritability: The effect of genetic factors can change during the life course, i.e., heritability can differ by age. For example, Silventoinen et al. (2007) analyze the Finish twin sample aged 16–25 years (*N* = 2,465 twin pairs) longitudinally: with increasing age the heritability of self-rated health decreases from 63% at age 16–33% at age 25—a value close the one reported by Røysamb et al. (2004). However, it is also possible that the effect of heritability increases with age; small contributions of genetic factors can cumulate over time and hence, have an increasing phenotypic effect. A study comparing a German (*N* = 4,092 twin pairs, aged 5–24 years) and U.S. twin sample (*N* = 1,886 twin pairs, aged 11–24 years) finds that in the German sample, the proportion of variance in BMI that genetic factors can explain increases with age (from 67% at age 5–82% at age 24) (Johnson et al., 2019). Common environmental factors vanish after the age of 11 and unique environmental factors explain the remaining variance. Also, in the U.S. sample, common environment cannot explain any part of the variance after age 11. Contrary to the results of the German sample, genetic factors decrease slightly with age (from 90% at age of 11 years−82% at age of 24). Interestingly, although the dynamics go in different directions, genetic factors are more important than environmental factors in both countries (Johnson et al., 2019). The result that importance of common environment vanishes at a certain age is also supported by Silventoinen et al. (2007).

The theoretical discussion of potential changes in the relationship of parental resources and children's health during childhood is still ongoing. Referring to Chen et al. (2002), the relationship between parental resources and health could either remain constant (*childhood-adolescent persistent model*), decrease (*childhood-limited model*) or increase (*adolescent-emergent model*) during childhood (Chen et al., 2002, p. 298–299).

In line with the above mentioned models by Chen et al. (2002), several empirical studies have analyzed the dynamic of the relationship of parental resources and children's health over time. On the one hand, in accordance with the childhood-adolescence persistent model (Chen et al., 2002, p. 298–299), Reinhold and Jürges (2012) as well as Nakamura (2014) show that the association of income and child health stays constant during childhood. On the other hand, some researchers find, in line with the adolescent-emergent model (Chen et al., 2002, p. 298), that the association of parental income and children's health increases as children grow older (Currie and Stabile, 2002; Khanam et al., 2009), whereas still others do not support this finding (West and Sweeting, 2004; Currie et al., 2007; Propper et al., 2007).

## 3. Research question and hypotheses

Our theoretical considerations as well as the empirical studies we report suggest that parental resources and genetic heredity both contribute to children's health. To our knowledge no study specifically examined the relative contribution of both factors and their interplay on children's health simultaneously.

**Figure 1:**
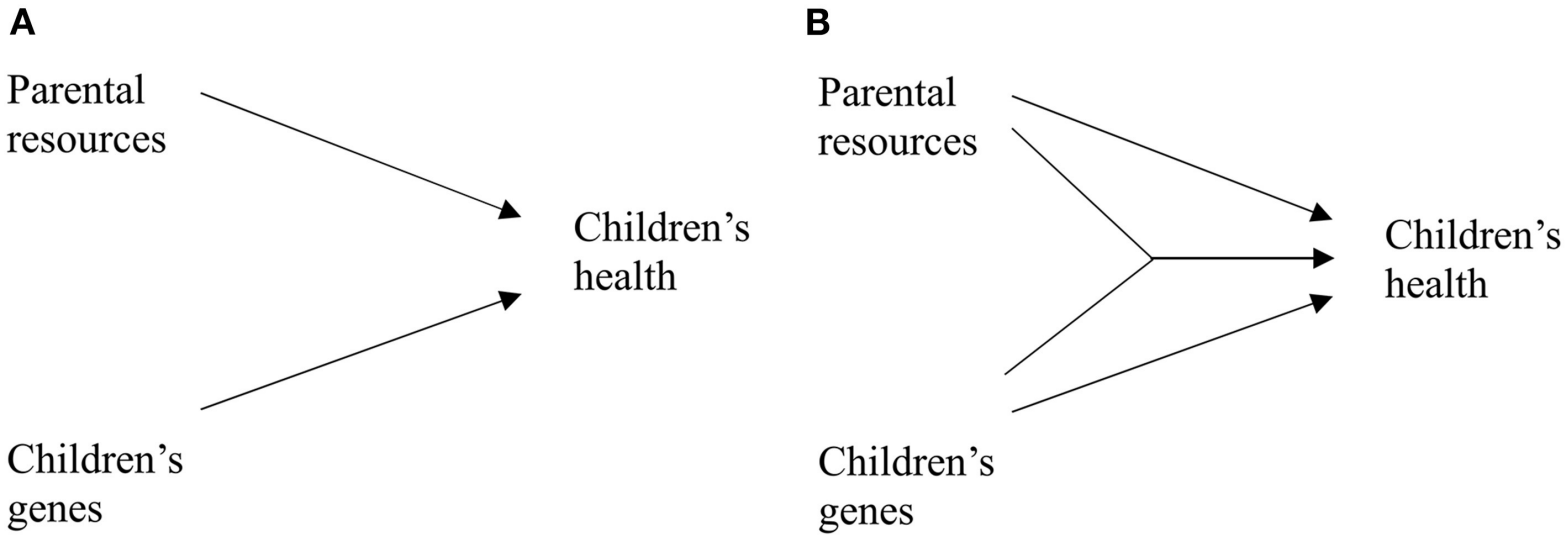
Relationship of parental resources, children's genes and children's health. **(A)** Additive effects. **(B)** Interaction effect.

We address this gap in the literature and analyze how much of the variance in children's health can be explained by parental resources and how much by genetic factors. We assume that parental resources and children's genetic endowments contribute to children's health (Figures 1A, B).

**Figure 2:**
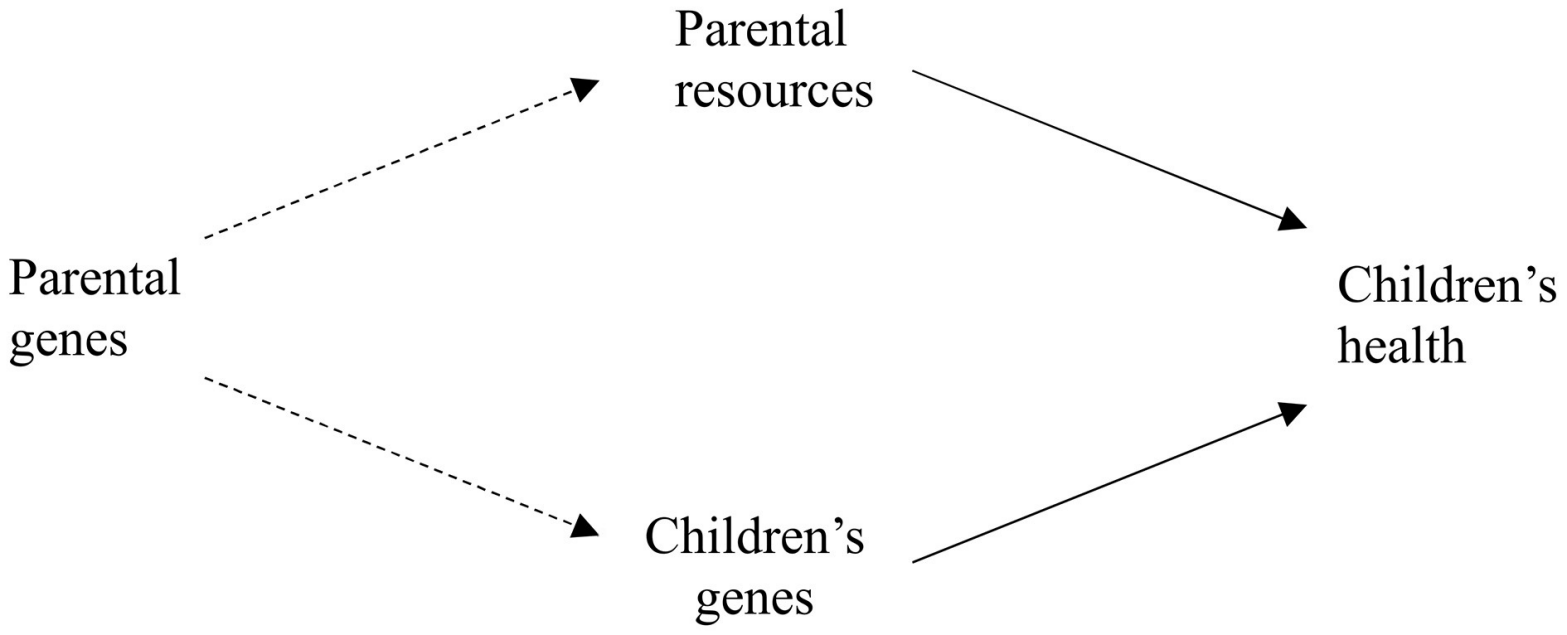
Parental genes affect both, parental resources and children's genes.

Some authors have argued that the relationship of parental resources and children's health could be caused by unobserved heterogeneity, in particular by parental genetic makeup (e.g., Propper et al., 2007; Johnson et al., 2019). In this case, parental genes would contribute to both, their resources, which in turn affect children's health and to their children's genes, which in turn also affect children's health (see Figure 2). However, we do not expect parental genes to affect child health directly. Hence, omitting parental genes does not lead to an omitted variable bias if child's genes are taken into account and the model in Figure 1 remains valid. Consequently, we focus on studying the joint effects of parental resources and children's genes on children's health.

To analyze how much of the variance in child health can be explained by parental resources and how much by genetic factors, we break down our research question into six specific hypotheses. These regard the association of parental resources and children's genes with children's health and how these associations might change during childhood and differ across status groups. The first hypothesis is:

**H1** The more resources parents of a child have the better the child's health.

Previous research has used different indicators to reflect parental resources: education (e.g., Ross and Mirowsky, 2011), occupation (Huurre et al., 2003), income (e.g., Nakamura, 2014) or maternal and paternal employment status (Nakamura, 2014; Jones, 2018). Most studies focus on mainly one of the above indicators but control for other dimensions of parental resources. Additionally, only few studies differentiate between mother's and father's resources although it seems plausible to assume that their effect on children's health differs. To shed light on these questions we will study the impact of education, employment status and income on children's health separately and distinguish between resources coming from fathers and mothers.

For twins reared together we assume that parental resources are part of the common environment of health and, thus, explain part of the variance subscribed to the common environment:

**H2** Parental resources explain part of the common environmental variance in child health.

We further assume that parental resources moderate the effect of heritability, as depicted in Figure 1B. In line with the results reported by Johnson et al. (2010), we assume that with increasing parental status the importance of the environment increases whereas the importance of genetic factors decreases. Accordingly, we formulate the following hypothesis:

**H3** With higher parental resources the part of variance in children's health explained by genetic factors decreases.

We further assume that if a family has more resources these offer twins more choices leading to more distinct personal, unique environments compared to families with less resources. We therefore hypothesize:

**H4** The more resources parents have, the higher the impact of the unique environmental variance on child health.

Finally, we are interested in how the gene-environment interplay develops during childhood. Empirical studies do not offer clear-cut results in this respect. Some authors find increasing genetic contribution to variance in health (e.g., Garcia et al., 2013; German sample Johnson et al., 2019), whereas others find decreasing effects (e.g., U.S. sample Silventoinen et al., 2007; Johnson et al., 2019). As the German sample used by Johnson et al. (2019) is the only one among the studies discussed above that covers the entire childhood period we posit that their findings will also hold for our study:

**H5** With increasing age, the variance in child health explained by genetic factors increases.

It also remains unclear how the relationship of parental resources and child health changes during childhood and adolescence. Theoretically, it is possible that the relationship increases, decreases or remains stable (compare Chen et al., 2002; Plomin et al., 2016). Empirical results of non-twin samples show no clear result (see for instance, Currie and Stabile, 2002; Currie et al., 2007; Propper et al., 2007; Khanam et al., 2009; Reinhold and Jürges, 2012). Twin studies indicate that the share of common environmental factors explaining variance in health decreases with increasing age indicating that the effect of parental resources as part of this common environment diminishes (e.g., Johnson et al., 2019). As twins grow older, they become more and more autonomous, both from each other and from their parents. Accordingly, with increasing age, the variance in child health explained by unique environment should increase. Consequently, our sixth hypothesis is:

**H6** With increasing age, the effect of the common environment on child's health decreases while the effect of the unique environment increases.

## 4. Data and analytical approach

### 4.1. Data

We use the first wave of the German Twin Family Panel, TwinLife (Diewald et al., 2020; GESIS data archive number ZA6701, version 5.0.0, https://doi.org/10.4232/1.13747), the first representative German twin family study. The study is based on a cohort-sequential design: beginning in 2014, TwinLife follows four cohorts of same-sex MZ and DZ twin pairs, their parents, the sibling closest to the twins, and partly, twins' partners. The study is conducted as a face-to-face interview every two years and as an alternating telephone interview every other year. TwinLife is based on a national probability sample randomly selected from Germanys' population registers initially covering 4,091 twin pairs (for details see Mönkediek et al., 2019; Lang and Kottwitz, 2020; Lang et al., 2020).

A comparison of TwinLife with the Microcensus, a yearly 1%-sample of the German population, showed that TwinLife is partially selective, especially in terms of education and German citizenship. A higher share of parents in TwinLife has tertiary education than parents in the general German population (Lang and Kottwitz, 2020). We therefore control for education and citizenship or migration background in all multivariate analysis. Additionally, we follow the suggestion by Lang and Kottwitz (2020) and include weighted analysis as a robustness check to ensure that the results of this study are representative (see Appendix Tables A2a, A2b, A3).

To limit unobserved heterogeneity of contextual characteristics in our sample we constrain it to those twin pairs up to the age of 18 living at home together with both of their biological parents. After excluding missing observations and incomplete twin pairs, the final sample size for analysis results in 3,168 twins (*N* = 1,584 twin pairs) (see Appendix Figure A1, for an overview of exclusion criteria and sample sizes). 21% of the twins report excellent health, 72% very good or good health and only 7% report less good or poor health (see Appendix Table A1a).

### 4.2. Operationalization

#### 4.2.1. Dependent variable: self-rated health

The dependent variable is twins' self-rated health, an item based on the Short Form Health-8 Survey (Ellert et al., 2005). Self-rated health is assessed using the question “How would you describe your state of health during the last 12 months, in general?” with six answer categories ranging from very poor (1) to excellent (6). Less than one percent of the twins rate their health as very poor, hence, we combine categories 1 (very poor) and 2 (poor) into a single category. For twins aged 6 years and younger health was parent-rated.

#### 4.2.2. Independent variables: parental resources

We operationalize parental resources as parental education and income. We include parental education as years of education based on information on general and vocational education, following the procedure by Baier and Lang (2019, p. 152). We calculate years of education separately for mothers and fathers. We also include parental income operationalized as logarithmic net equivalized household income accounting for household size and composition. We use the logarithm of income because it reflects relative rather than absolute income differences (Verbeek, 2012, p. 84) and is robust against outliers. We also create a variable reflecting socioeconomic status (SES) of the household based on mothers' and fathers' years of education and income using principal component analysis. Combining these indicators into a single SES measure is not only motivated by theoretical considerations (see above) but may be advisable from a statistical perspective as well. Though the correlations between the three status measures are not exceedingly high^2^ their mutual correlations may cause estimation problems due to increased multicollinearity in the regression analyses.

Finally, we include indicators for employment status of fathers and mothers based on the empirical findings by Jones (2018) and Nakamura (2014) reported above. These variables reflect mothers' and fathers' available time for childcare. For fathers this is a dummy variable indicating if a father is working full-time or not. Since mothers often work part-time, we specify maternal employment status as a three-level variable differentiating not working, working part-time and working full-time, respectively.

#### 4.2.3. Control variables

To reduce unobserved heterogeneity and because of the cross-sectional design of our study we include control variables which have been shown to affect either children's health or children's health and parental resources simultaneously. We control for twin pairs' zygosity (0 = dizygotic; 1 = monozygotic), twins' sex (0 = female; 1 = male) and twins' age. We expect to affect both twins' self-rated health and parental resources. First, we include a dummy variable conditioning on migration background of twins and their families (0 = born in Germany; 1 = twins or at least one of their parents were not born in Germany or do not have German citizenship). On the one hand, migrants tend to have lower education, occupational qualifications and income than non-migrants, sometimes due to nonrecognition of their degrees or language barriers. Similarly, language difficulties as well as limited knowledge of the German healthcare system can limit access to healthcare for migrants (Steinbach, 2018). On the other hand, migrants tend to have better health due to positive health-related self-selection (healthy-migrant effect, Spallek and Razum, 2008, p. 277). Next, we control for regional characteristics with two variables. First, we include a dummy variable indicating whether the twins live in former East (0) or West (1) Germany. Despite a decrease since German reunification in 1990, differences in important spheres of life such as life expectancy and health (see Prütz et al., 2014 for an overview) but also in income, education and employment status remain (BMAS, 2021). Second, we include a variable indicating the city size of the twin families' place of residence. Access to healthcare and health prevention, environmental conditions such as pollution or access to green spaces as well as health behavior can differ between urban and rural areas (see Weidmann and Reime, 2021 for an overview) which again can affect children's health. Also, parental resources can differ by area of living: high-skilled jobs are often concentrated in urban areas. Moreover, educational opportunities and income differ between urban and rural areas (Hirsch et al., 2009; Weishaupt, 2010, p. 226 - 227). Appendix Tables A1a, A1b gives an overview of all variables and their distributions. Our analyses are based on the restricted sample of complete cases which do not differ greatly from the full sample. We mean-center all continuous variables.

### 4.3. Methods

#### 4.3.1. OLS regression

We test our first hypothesis that children with more parental resources have better health with OLS regressions because the results are easy to understand. We did, however, also run these analyses with ordered logit regressions to see if they stand up when easing the assumption on measurement level of the health variable. The substantive conclusions remain unchanged (see Appendix Tables A2a, A2b). A particularity of the present data is its dyadic structure (Kenny et al., 2006). We follow the suggestion by Aronow et al. (2015) and include cluster robust standard errors to account for the present dyadic structure. Other ways to treat such data are multilevel models which we also ran (see Appendix Tables A2a, A2b). Finally, we report analyses using complete cases, i.e., excluding all cases with missing values (see above for sample selection criteria). To test if this restriction of the sample biases results, we also ran all analyses with structural equation models using full information maximum likelihood estimates for the full sample (see Appendix Tables A2a, A2b). We repeated all these analyses using a redressment weight aiming to correct for possible nonresponse bias. Neither of these robustness checks led to any divergent results.

#### 4.3.2. Variance decomposition

To analyze hypotheses 2–6, we decompose the variance in self-rated health by using a *Classical Twin Design* (CTD). CTD is a common way to disentangle the contribution of genetic and environmental influences on a phenotype (Purcell, 2013, p. 392–393; Rijsdijk and Sham, 2002). It takes advantage of the fact that MZ twins share 100 percent of their genes, whereas DZ twins share, on average, 50 percent of their genes. If we assume that twins raised by the same parents grow up in the same environment (Hatemi et al., 2010), this allows to decompose the variance of a phenotype into genetic [*additive* genetic factors (A)]^3^ and environmental factors (*common* environment (C) and *unique* environment (E)). Additive genetic effects (A) are the main effects of genetics on the outcome. Common environmental factors (C) describe the influences affecting both twins such as socioeconomic status or place of residence. These factors are characteristics that make twins reared by the same parents more alike than twins growing up separately. Unique environmental factors (E) are the individual circumstances that lead twins to be less similar such as different circles of friends or different hobbies. Another part of unique environmental factors is measurement error (Plomin et al., 2013). The variance decomposition of an outcome variable into additive genetic effects (A), common environmental effects (C) and unique environmental effects (E) can be calculated in one model, the so-called ACE model.

ACE models are based on the following assumptions: First, the equal environments assumption states that differences between MZ and DZ twins do not exist because MZ and DZ twins are treated differently by their environments. This assumption is strongly discussed and questioned in the literature [see a summary of criticism by Horwitz et al. (2003)]. Others argue that only if differences in environments of DZ and MZ twins would affect the outcome under study, the assumption would be violated and genetic effects overestimated (Medland and Hatemi, 2009; Hatemi et al., 2010). Also, the evidence that MZ and DZ twins are treated differently seems empirically untenable (Conley et al., 2013). Accordingly, we rely on the assumption that differences in environment based on zygosity do not affect self-rated health.

The second assumption is assortative mating with respect to the outcome under study. Non-random parental mating in terms of self-rated health would result in a higher similarity of health within parents. This again increases the genetic similarity within a family (Plomin et al., 2013; Baier and Lang, 2019; Johnson et al., 2019) and the assumption that DZ twins share 50 percent of their genes would be violated (Hatemi et al., 2010, p. 803). Thus, genetic variance might be underestimated and common environmental factors overestimated (Plomin et al., 2013, p. 197–198). Given the information on parental health, it is possible to include a correction for assortative mating. The correction is based on


$$\begin{array}{l} 0.5\;+\;0.5\;*{h_{0}}^{2}*r_{p} \end{array}$$

where *h*${}_{0}^{2}$ describes the relative contribution of genetic factors without correcting for assortative mating and *r*_*p*_ describes the correlation of mother's and father's health, in our case 0.13 (Baier and Lang, 2019, p. 155; Loehlin et al., 2009, p. 166). After correcting for assortative mating, we obtain an expected genetic correlation for DZ twins equal−0.52.

Third, ACE models assume that different genes affect the outcome under study independently. This means that genetic effects are additive and that no genetic interactions influence the outcome under study (genetic correlation of DZ twins is 0.5) (Neale, 2009; Lang, 2017).

To test hypotheses 3–6 we use gene-environment interaction models that allow to analyze the interaction of environment and genetic factors (compare Purcell, 2002; Dinescu et al., 2016; Johnson et al., 2019). Therefore, the ACE model is modified by including a moderator variable that can be shared (as in our case) or differ between twins (Purcell, 2002). As specified by Bates et al. (2019) gene-environment interactions are often used to estimate changes in heritability moderated by, e.g., parental socioeconomic status. We estimate two sets of gene-environment interaction models, one treating parental resources as a moderator and one using twins' age.

We estimate the regression and ACE models in Stata 17, using acelong.ado (Lang, 2017) for the ACE variance decomposition. Acelong computes ACE variance decomposition based on a linear multilevel mixed-models parametrization (Rabe-Hesketh et al., 2008). We estimate the gene-environment interactions in R using the package umx (Bates et al., 2019).

## 5. Results

We begin our analysis by testing whether more parental resources are beneficial for children's health (H1). In addition, we explore if it makes a difference if a resource is held by mothers or fathers. Table 1 presents the analyses in which we regressed child health on several sets of parents' resources. In Models 1 through 3 we tested the effect of education of mother and father (1), the effect of household income (2) as well as the effect of mother's and father's employment status (3) on the health of twins, respectively. According to these results father's education, household income and father's employment status each are positively related to children's health. If we regress child health on all parental resources simultaneously the effect sizes of these variables diminish leaving father's education and income only barely statistically significant while employment status loses its significance (Model 4). If we control for possible confounders the picture changes slightly (Model 5). The effect of income disappears while the effect size for father's employment status increases and surpasses statistical significance (*p* < 0.1) again whereas father's education remains associated with children's health.^4^ These results are corroborated when using weighted results, when applying multilevel models or when using the full sample and full information maximum likelihood to estimate coefficients exploiting all available information in the data (see Appendix Table A2a).

**Table 1:** OLS regression, complete cases, no weights.

	**M1**	**M2**	**M3**	**M4**	**M5**	**M6**	**M7**
Mothers' years of education	0.010			0.009	0.005		
	(0.007)			(0.008)	(0.008)		
Fathers' years of education	0.017^*^			0.013^#^	0.016^*^		
	(0.007)			(0.007)	(0.007)		
Household income		0.082^**^		0.046^#^	0.041		
		(0.026)^**^		(0.027)^*^	(0.027)		
Parental SES						0.082^***^	0.074^***^
						(0.018)^**^	(0.019)^***^
**Mother employment**							
Part-time			0.050	0.010	0.037		0.034
			(0.044)	(0.045)	(0.045)		(0.044)
Full-time			0.051	−0.002	0.048		0.038
			(0.058)	(0.060)	(0.061)		(0.060)
Fathers' employment			0.123^#^	0.091	0.119^#^		0.124^#^
Full-time			(0.064)	(0.064)	(0.064)		(0.063)^*^
Monozygosity					0.073^*^		0.072^*^
					(0.036)		(0.036)^*^
Male twins					0.029		0.029
					(0.036)		(0.036)
Age twins, centered					−0.024^***^		−0.023^***^
					(0.004)^**^		(0.004)^**^
West Germany					0.090^#^		0.089^#^
					(0.053)^*^		(0.052)^*^
City size, centered					0.011		0.010
					(0.017)		(0.017)
Migration background					0.091^#^		0.089^#^
					(0.051)^*^		(0.051)^*^
Constant	3.791^***^	3.646^***^	3.789^***^	3.702^***^	3.515^***^	3.792^***^	3.519^***^
	(0.018)	(0.069)	(0.018)	(0.069)	(0.091)	(0.018)	(0.091)
Observations	3,168	3,168	3,168	3,168	3,168	3,168	3,168
Adjusted *R*^2^	0.007	0.002	0.004	0.008	0.026	0.008	0.027
*AIC*	8,119.213	8,137.332	8,128.561	8,119.252	8,066.620	8,113.323	8,063.771
*BIC*	8,137.395	8,161.575	814.683	8,161.678	8,145.411	8,125.444	813.440

Standardized coefficients; standard errors in parentheses, ^#^p < 0.1, ^*^p < 0.05, ^**^p < 0.01, ^***^p < 0.001.

So far, our results indicate that father's education and employment status are the instrumental resources shaping health of children. Because we also control for income, we assume that education does not reflect financial aspects but more cognitive ability, knowledge, cultural capital or “learned effectiveness” enabling people “to coalesce health-producing behaviors into a coherent lifestyle” (Mirowsky and Ross, 2003: p. 25).

As pointed out above we followed Link and Phelan (1995) and took a second look at the effect of inequality on health by combining education of mother and father as well as household income into a combined SES measure and repeated our analyses. The results—listed in Table 1, Models 6 and 7—show a consistent positive effect of household SES on health of children which is highly statistically significant.^5^

Comparing Models 5 and 7 we see that the effects of the other variables in the models are not affected by how we operationalize social resources of parents. We find a consistent negative age effect indicating the slow deterioration of children's health over time.^6^ Monozygotic twins, those living in West Germany and twins with a migration background have a slight advantage over dizygotic, East German or autochthon twins, respectively. Gender and size of place do not seem to be related to children's health.

To test our second hypothesis we estimate how much variance of twins' health is due to heritability, how much to common environmental conditions and how much to unique environmental conditions of each twin. To answer this question, we use ACE-models (Rijsdijk and Sham, 2002), which decompose the variance of an outcome variable in a sample of MZ and DZ twins into the three components: A: genetics, C: common environment, E: unique environment including measurement errors (see also Footnote 2). In all ACE models we correct for assortative mating. According to Model 1 in Table 2 almost a third of the variance of self-rated health can be attributed to heritability (A); the environment shared by the twins (C) accounts for 12% of the variance; while the environment unique to each twin (E) is responsible for over half of the variance.^7^

**Table 2:** ACE variance decomposition of twins' self-rated health, corrected for assortative mating, no weights.

	**Model 1**			**Model 2 SES as covariate**			**Model 3 SES and controls as covariates**		
	**var**	**se**	**%**	**var**	**se**	**%**	**var**	**se**	**%**
A	0.25	0.068	32.6	0.25	0.068	32.4	0.24	0.065	31.5
C	0.09	0.053	12.4	0.09	0.053	11.8	0.08	0.050	10.8
E	0.42	0.027	55.1	0.42	0.027	55.0	0.42	0.027	55.2
Total	0.76	0.021	100	0.76	0.021	99.2	0.76	0.021	97.5

Var, absolute variance; se, standard error of variance; %, variance per component in percent of total variance. N = 3,168.

In our second hypothesis we argued that parental resources are part of the common environment of twins and should therefore account for some of this variance. Indeed, if we introduce SES as a composite score reflecting parental resources, we observe a very slight decrease in variance component C (cf. Model 2 in Table 2). Though this change is consistent with hypothesis 2 we hesitate to interpret it as confirming our expectation due to its very small size. If we introduce all control variables used in the regression analysis above the share of common variance decreases further (Model 3 in Table 2).^8^

**Figure 3:**
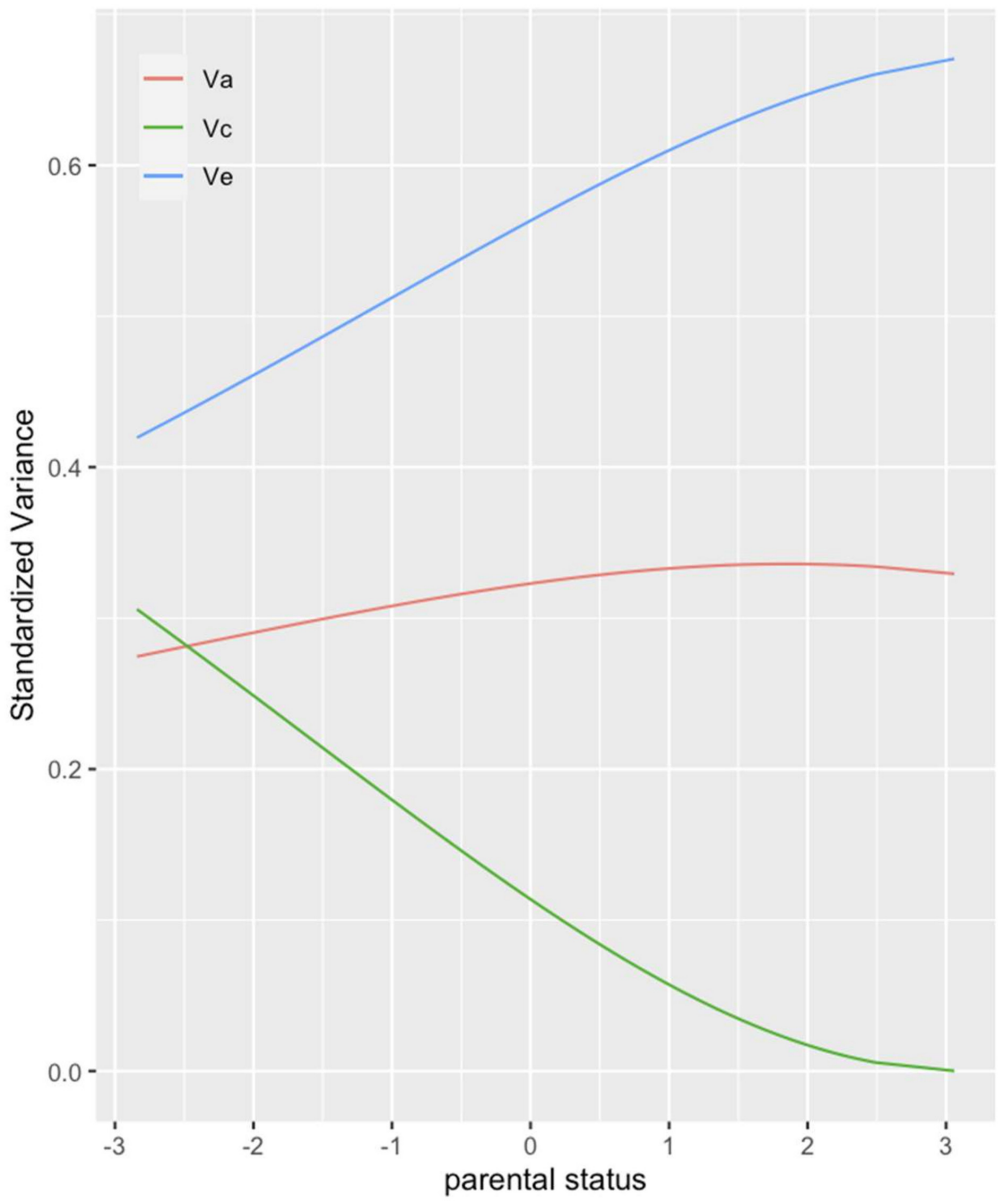
ACE decomposition by parental status (parental SES). The figure presents the share of variance in twins' health that genetic (red), common environmental (green) and unique environmental (blue) factors can explain. Corrected for assortative mating, no weights.

In our third hypothesis we express our expectation that the part of variance in children's health explained by genetic factors decreases with higher parental resources. We again use ACE models to test this hypothesis, this time with parental SES as moderator variable. Figure 3 displays the standardized moderation effects of status on common environment (C), unique environment (E) and genetic factors (A). With increasing SES the impact of genetic factors on health increases slightly from about (28–33%). This contradicts the results by Johnson et al. (2010, 2019), who found that variance in health explained by genetic factors decreases with increasing status. Instead, in our analysis the role of genetic factors on the formation of health tends to increase slightly with increasing parental status. However, Figure 3 also shows that the moderation of status on environmental factors is stronger than on genetic factors. In line with our fourth hypothesis, the part of variance explained by the environment unique to each twin increases with increasing parental status. Thus, the unique environment plays a more important role on the formation of children's health in families with higher parental status than in families with lower parental status. Accordingly, the part of variance in health explained by the environment shared by the twins (common environment) decreases with increasing parental status and plays almost no role at all for twins with the highest SES family background. The results of the moderation of status on common and unique environment supports our idea that higher parental status provides the twins more opportunities leading to more differentiated unique environments.

**Figure 4:**
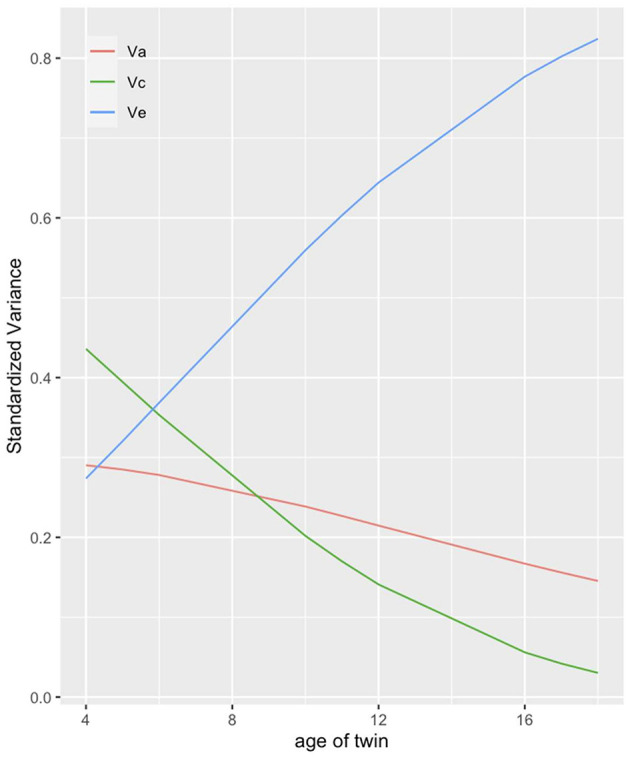
ACE decomposition by age of twin. The figure presents the share of variance in twins' health that genetic (red), common environmental (green) and unique environmental (blue) factors can explain. Corrected for assortative mating, no weights.

Finally, we are interested in how heritability and environmental conditions shape children's health during the first 18 years of their lives. We assumed that genetic factors become more important (H5) as well as the unique environment while the common environment should lose its importance at higher ages (H6). To test these hypotheses, we conduct an ACE model using twins' age as moderator. In line with Silventoinen et al. (2007) and the U.S. sample by Johnson et al. (2019), genetic factors explaining variance in health decrease with increasing age, contradicting our fifth hypothesis stating the opposite (Figure 4). Just as the impact of genetic factors decreases with age so, too, does the impact of the common environment. In contrast the variance explained by the unique environment increases with age. This supports the reasoning that twins become more and more autonomous with increasing age, which in turn has an increasing impact on the formation of their health.

## 6. Discussion and conclusion

In this article we used twin data to examine the impact of parental resources and heritability on children's health. Based on theoretical considerations and previous research we formulated six hypotheses expressing our expectations concerning the impact of parental resources, genetic predispositions and their interplay on health of children.

Overall, self-rated health of the twins is good to very good, only 7% state that their health is less than good. This puts the proportion of those with not good health in a similar range to that reported by Kuntz et al., 2018). In accordance with our expectations, and as shown in previous research (e.g., Reinhold and Jürges, 2012; Nakamura, 2014; Kuntz et al., 2018), we found that parental resources positively affect health of their offspring (H1). When differentiating between different types of resources provided by mothers and fathers, we found a strong and consistent effect of father's education. Possible pathways linking father's education to child's health could, for example, be that families with well-educated fathers are characterized by healthy lifestyles including basic health education for their children. This finding is similar to the results by Jones (2018) who also find a strong association of father's education and children's weight. We also expected that parental resources explain part of the environmental influence that twins share with each other (H2). Because parental resources only explain a very small part of variance in children's health controlling for them does only change the estimates for variance components slightly.

Not confirmed was our third hypothesis in which we stated that genetic factors should become less important in shaping children's health with increasing SES of parents. Instead, we found a small increase in the impact of heritability for higher compared to lower SES families, contradicting the results by Johnson et al. (2019) who found a decrease of genetic influence with higher parental SES on BMI. But SES clearly moderated the impact of common and unique environments. While for low SES families the environment shared by children was almost as important as the child-specific environment, for families with high SES common environment lost its impact almost entirely. In contrast, unique environment showed the strongest effect on child's health. This finding demonstrates how resources offer choices and give children of the same family the opportunity to develop their own “environments”, e.g., pursue different leisure time activities, choose different groups of friends etc. which in turn differentially influence their health. Finally, we assumed that the impact of heritability increases with age during childhood and adolescence (H5). This expectation was not confirmed, similar to the results by Johnson et al. (2019) for the U.S. sample. Additionally, we saw that also the impact of the common environment loses its meaning for health while growing up thereby increasing the importance of environmental factors unique to each twin (H6). This is in line with the results by Silventoinen et al. (2007) and Johnson et al. (2019).

Our study focusses on children aged 4–18 in Germany. As stated by Plomin et al. (2013) different environments (e.g., other country) may alter the relative contributions of environment and genetics. Without empirical data it is difficult to assess whether our results are specific for Germany or whether they would also hold for other countries. In an analysis of socioeconomic and hereditary factors of BMI comparing Germany with the USA Johnson et al. (2019) report similar findings for both countries though results differ in some details. With respect to the research questions studied by us we would assume that our general conclusions would also hold for countries with similar welfare and healthcare systems.

### 6.1. Limitations

The main advantage of twin studies, compared to regular surveys, is that they allow to consider genetic factors in addition to environmental factors. This strength, however, is combined with certain weaknesses: Twin pregnancies are often high-risk pregnancies, compared to singletons, twins are born 3 weeks earlier on average and have lower birth weight, twins also have a higher risk for poor health (Lytton and Gallagher, 2002, p. 229). In addition, it could be that the probability for twin pregnancies depends on genetic disposition, which may also affect health. Some authors therefore question if results of twin studies can be transferred to non-twin families (Sahu and Prasuna, 2016). Empirical studies, however, have repeatedly demonstrated that results on heritability from twin studies could be corroborated with other designs (see Hatemi et al., 2010).

Although the operationalization of health as self-rated health is one strength of this study, the overall variance is comparatively low. It could be that other operationalizations of health with more variance would achieve other results. TwinLife provides mental health measures, however, these items are only surveyed from the second wave on and not available for the youngest children. Due to the relatively high drop out of participants between waves, we decided to use the first wave to maximize sample size. Further, strict inclusion criteria of our sample as well as deletion of missing cases (see Appendix Figure A1) could bias results. However, robustness checks using all eligible cases including those with missing values confirm the conclusions reported above.

For children up−6 years, information on health was provided by parents; all older children self-report. Therefore, we cannot fully exclude the possibility of bias in the data due to potential differences in response behavior. Parental assessment of twins' health could, for example, either lead to more similar or to a greater differentiation of health estimates compared to self-assessment. To our knowledge no empirical study directly investigated the agreement of self-rated health reported by parents and children. We would, however, expect a high degree of consistency between self- and proxi-reports since the measure is easy to understand and straightforward.

As mentioned earlier, it would be interesting to analyze the role of children's own resources in comparison to parental resources on health. A study on a national representative U.S. sample finds that own education can buffer the effect of low parental education on health (Ross and Mirowsky, 2011).

### 6.2. Conclusion

Our study confirms the consistent effect of social inequality in terms of parental resources on children's health. We were interested in disentangling the effects of different aspects of SES and different sources of resources by analyzing education and employment status of mothers and fathers as well as household income separately. The results showed that among the resource indicators we were able to analyze father's education and to a lesser extent his involvement in the labor market had substantial effects on children's health.

When decomposing the variance of health with the help of ACE models we found that about a third of health's variance is determined by genetic factors, just over 10 percent by environmental factors common to both twins and over half of the variance by environmental characteristics unique to each twin. We found clear evidence that the influence of heritability and environmental factors vary by SES and by age of children. This finding demonstrates that the question of “nature vs. nurture” is not a simple one but depends on many circumstances and varies between individuals living in different social circumstances.

Although parental resources may explain some of the variance in children's health, this proportion is relatively small. Therefore, when adapting policy recommendations, the focus should also be on other factors that influence children's health. A large part of children's health can be explained by the unique environment, so a next step could be to investigate which factors exactly belong to the unique environment. Then, interventions designed to reduce the inequitable distribution of health in children could link to these factors. A closer look at the unique environment is also interesting in that as children and adolescents grow older, the influence of the unique environment on health increases as their own resources become more important.

## Data availability statement

Publicly available datasets were analyzed in this study. This data can be found here: GESIS Data Archive; https://search.gesis.org/research_data/ZA6701.

## Ethics statement

Study was approved by Ethic Committee of the German Psychological Society in 2010 and again in 2013. Written informed consent to participate in this study was provided by the participants' legal guardian/next of kin.

## Author contributions

BH and CW contributed to conception and design of the study, performed the statistical analysis, and wrote sections of the manuscript. BH wrote the first draft of the manuscript. All authors contributed to manuscript revision, read, and approved the submitted version.

## Footnotes

^1^Let us assume *h*^2^ is the variance of a trait caused by heritability, *c*^2^ is the variance caused by common environmental factors and the contribution of the environment unique to each twin is *e*^2^. Then a simplified model for the correlation of a trait within an MZ and DZ twin pair is: $r_{MZ}=h^{2}+c^{2}$ and $r_{DZ}=\frac{h^{2}}{2}+c^{2}$. This gives $h^{2}=2\left(r_{MZ}-r_{DZ}\right)$ and $c^{2}=r_{MZ}-h^{2}$. If we further assume that *h*^2^+*c*^2^+*e*^2^ = 1 this gives $e^{2}=1-r_{MZ}$ (for more details see Purcell, 2013: 381-383).

^2^Our SES measure consists of three indicators: father's education, mother's education, and household income. Multiple correlation of father's education with the other two status measures is 0.56; for mother's education with the other two measures, it is 0.57 and the multiple correlation for income with the two educational measures is 0.31.

^3^The variance of a phenotype can also be decomposed into d*ominant* genetic factors, which in turn are genetic interactions. Given the following rule of thumb, the genetic factors explaining variance in twins' self-rated health are not dominant; hence, we do not further address dominant genetic factors in this work. If the correlation of a trait among DZ twins is at least half the correlation among MZ twins (r_DZ_ ≥ 0.5^*^r_MZ_) genetic factors should be treated as *additive* genetic factors. If r_DZ_ < 0.5^*^r_MZ_, genetic factors should be treated as *dominant* genetic factors (Rabe-Hesketh et al., 2008; Medland and Hatemi, 2009; Hatemi et al., 2010; Baier and Lang, 2019). Because in the present sample the correlation of DZ twins' health is larger than 0.5 times the correlation of MZ twins' health (0.22 compared to 0.30) we use ACE models.

^4^We tested all models for collinearity; the largest variance inflation factor found for Models 1 through 7 in Table 1 was 1.73 for mother's education in Model 5, way below any critical value.

^5^This result is unchanged if we use weighted data or different estimation methods (see Appendix Table A2b).

^6^We did not find any non-linear age effects.

^7^One could argue that the large share of “unique” environment to a large part reflects measurement error of self-rated health because it might be difficult to measure this concept in children. However, as readers will see in a later analysis (Figure 2) a decomposition of health differences by age shows that the share of variance attributed to “unique” environment *grows* with age. Assuming that measurement error is smaller in older than younger children this indicates that E most likely does not (only) reflect measurement error.

^8^As can be seen from Appendix Table A3 results based on weighted cases are not substantively different although the results are more in line with hypothesis 2: controlling for SES and the other covariates consistently decreases the variance attributed to the common environment of twins.
