# Supplementary material for: Parental resources and heritability as factors shaping children's health. An analysis of twins' self-rated health using TwinLife
Source: Front Sociol. 2023 Jun 27;8:1136896. doi: 10.3389/fsoc.2023.1136896 (PMC10333594; doi:10.3389/fsoc.2023.1136896)
Supplement: Supplementary file 1 [file Data_Sheet_1.pdf]

Supplementary Material

Parental Resources and Heritability as Factors Shaping Children's Health. An Analysis of Twins' Self-rated Health using TwinLife

Bärbel Holzwarth, Christof Wolf*

*** Correspondence:** Christof Wolf, ORCID 0000-0002-9364-9524, christof.wolf@gesis.org

Supplementary figures and tables

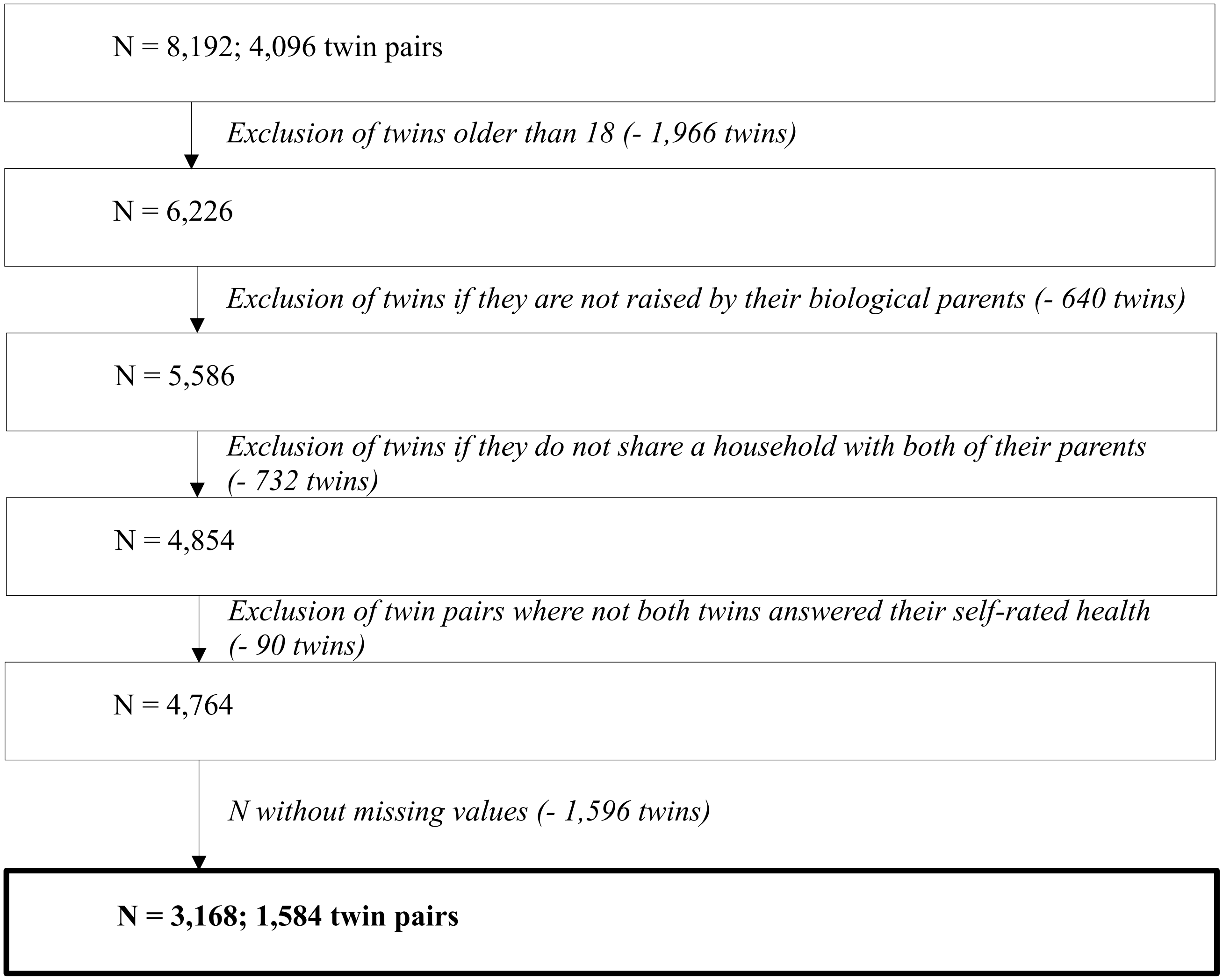

Figure A1: Flowchart of exclusion criteria and deletion of missing values. Boxes display sample size of the initial sample and after each step of excluding cases and data preparation. Italic phrases explain each step of exclusion and preparation in detail

Table A1a: Descriptive statistics for dependent and independent variables used in analyses by full sample and restricted sample (complete cases)

	Full sample		Complete cases	
**Categorical Variables**				
	**N**	**%**	**N**	**%**
Twins' health[a]	4,764	100.0	3,168	100.0
1: poor	54	1.1	32	1.0
2: less good	296	6.2	186	5.9
3: good	1,269	26.6	856	27.0
4: very good	2,066	43.4	1,426	45.0
5: excellent	1,079	22.7	668	21.1
Employment status mother	4,492	100.0	3,168	100.0
1: inactive or unemployed	1,238	27.6	850	26.8
2: working part-time	2,476	55.1	1,776	56.1
3: working full-time	778	17.3	542	17.1
Employment status father	3,738	100.0	3,168	100.0
0: other than full-time	404	10.8	330	10.4
1: working full-time	3,334	89.2	2,838	89.6

	Full sample			Complete cases		
**Continuous Variables**						
	**N**	**Min Max**	**Mean (SD)**	**N**	**Min Max**	**Mean (SD)**
Twins' health[a]	4,764	1<br>5	3.80<br>(0.90)	3,168	1<br>5	3.79<br>(0.87)
Years of education mother	4,528	7<br>20	14.09<br>(3.10)	3,168	7<br>20	14.13<br>(3.04)
Years of education father	3,764	7<br>20	14.34<br>(3.27)	3,168	7<br>20	14.45<br>(3.22)
Net household equivalent income[b] in €	4,212	115<br>22,957	1,807<br>(1,410)	3,168	123<br>22,957	1,875<br>(1,484)

[a] We present self-rated health as both, categorical and continuous variable.
[b] Table A1a displays the regular household net equivalent income. For further analyses, we include the logarithmic household net equivalent income.

Table A2a: Model 5 from Table 1 estimated with different methods, without and with weights

	OLS	OLS/w	OLM	OLM/w	MLM	MLM/w	SEM	SEM/w
Mothers' years of education	.005	.008	.013	.019	.005	.006	.008	.010
	(.008)	(.008)	(.017)	(.018)	(.008)	(.008)	(.007)	(.008)
Fathers' years of education	.016*	.017*	.033*	.034*	.016*	.016*	.014*	.013#
	(.007)	(.008)	(.015)	(.016)	(.007)	(.007)	(.007)	(.007)
Mother employment								
part-time	.037	.021	.066	.030	.037	.029	-.054	-.050
	(.045)	(.050)	(.098)	(.108)	(.043)	(.046)	(.058)	(.065)
full-time	.048	.038	.117	.092	.048	.042	-.056	-.041
	(.061)	(.066)	(.132)	(.139)	(.059)	(.062)	(.042)	(.048)
Father employment full time	.119#	.092	.253#	.175	.119*	.105	.117#	.084
	(.064)	(.080)	(.137)	(.169)	(.060)	(.070)	(.061)	(.075)
Household income	.041	.035	.105#	.094	.041	.040	.041	.036
	(.027)	(.031)	(.059)	(.066)	(.028)	(.028)	(.027)	(.030)
Monozygosity	.073*	.079#	.161*	.175*	.073*	.080*	.069*	.069#
	(.036)	(.042)	(.078)	(.089)	(.036)	(.038)	(.034)	(.039)
Male twins	.029	.060	.045	.094	.029	.046	.039	.067#
	(.036)	(.041)	(.078)	(.088)	(.036)	(.037)	(.034)	(.039)
Age twins	-.024***	-.022***	-.047***	-.042***	-.024***	-.023***	-.025***	-.024***
	(.004)	(.004)	(.008)	(.009)	(.004)	(.004)	(.003)	(.004)
West Germany	.090#	.099#	.194#	.211#	.090#	.094#	.085#	.091#
	(.053)	(.056)	(.116)	(.123)	(.053)	(.053)	(.049)	(.052)
City size	.011	.004	.004	-.011	.011	.009	.005	-.002
	(.017)	(.020)	(.038)	(.042)	(.017)	(.018)	(.016)	(.018)
Migration background	.091#	.100#	.214#	.224#	.093#	.104#	.083#	.096#
	(.051)	(.059)	(.111)	(.126)	(.050)	(.054)	(.049)	(.057)
Constant	3.515***	3.521***			3.515***	3.516***	3.629***	3.627***
	(.091)	(.107)			(.086)	(.096)	(.089)	(.101)
Observations	3168	3168	3168	3168	3168	3168	3498	3498
Adjusted R^2	.026	.025						
AIC	8066.620	8154.410	7888.333	7141.763	7878.311	7091.140	1.0e+05	9.3e+04
BIC	8145.411	8233.201	7985.307	7238.736	7969.224	7182.053	1.0e+05	9.4e+04

OLS models, ordered logit models (OLM), multilevel models (MLM), structural equation models (SEM) estimated with full sample using Full Information Maximum Likelihood estimation. Standard errors in parentheses, # $p < .1$, * $p < .05$, ** $p < .01$, *** $p < .001$; weighted models marked /w.

Table A2b: Model 7 from Table 1 estimated with different methods, without and with weights

	OLS	OLS/w	OLM	OLM/w	MLM	MLM/w	SEM	SEM/w
Parental SES	.074***	.080***	.168******	.181***	.074***	.077***	.074***	.080***
	(.019)	(.020)	(.042)	(.044)	(.019)	(.019)	(.019)	(.020)
Mother employment								
part-time	.034	.019	.060	.026	.035	.027	-.050	-.047
	(.044)	(.050)	(.097)	(.107)	(.043)	(.046)	(.057)	(.063)
full-time	.038	.032	.098	.079	.038	.034	-.055	-.040
	(.060)	(.064)	(.130)	(.136)	(.058)	(.061)	(.042)	(.048)
Father employment full time	.124#	.094	.264#	.180	.124*	.109	.121*	.085
	(.063)	(.079)	(.136)	(.168)	(.059)	(.069)	(.060)	(.074)
Monozygosity	.072*	.079#	.159*	.174*	.072*	.079*	.069*	.070#
	(.036)	(.042)	(.078)	(.088)	(.036)	(.038)	(.034)	(.039)
Male twins	.029	.060	.044	.093	.029	.046	.037	.065#
	(.036)	(.041)	(.078)	(.087)	(.035)	(.037)	(.033)	(.039)
Age twins	-.023***	-.022***	-.047***	-.042***	-.023***	-.023***	-.024***	-.023***
	(.004)	(.004)	(.008)	(.009)	(.004)	(.004)	(.003)	(.004)
West Germany	.089#	.098#	.194#	.210#	.088#	.093#	.085#	.092#
	(.052)	(.056)	(.116)	(.122)	(.053)	(.053)	(.049)	(.052)
City size	.010	.004	.003	-.011	.010	.009	.005	-.002
	(.017)	(.019)	(.038)	(.042)	(.017)	(.018)	(.016)	(.018)
Migration background	.089#	.101#	.208#	.223#	.091#	.103#	.081	.096#
	(.051)	(.059)	(.110)	(.125)	(.050)	(.054)	(.048)	(.056)
Constant	3.519***	3.526***			3.519***	3.520***	3.626***	3.627***
	(.091)	(.107)			(.086)	(.096)	(.088)	(.100)
Observations	3168	3168	3168	3168	3168	3168	3498	3498
Adjusted R^2	.027	.025						
AIC	8063.771	8151.037	7885.340	7138.155	7875.174	7087.673	9.0e+04	6.6e+04
BIC	813.440	8217.707	7970.192	7223.007	7953.965	7166.464	9.0e+04	6.6e+04

OLS models, ordered logit models (OLM), multilevel models (MLM), structural equation models (SEM) estimated with full sample using Full Information Maximum Likelihood estimation.
Standard errors in parentheses, # $p < .1$, * $p < .05$, ** $p < .01$, *** $p < .001$; weighted models marked /w.

Table A3: ACE variance decomposition of twins' self-rated health corrected for assortative mating, weighted

	Model 1			Model 2 SES as covariate			Model 3 SES & controls as covariates		
	Var	se	%	var	se	%	var	se	%
A	.18	.071	23.7	.18	.072	24.1	.18	.068	24.3
C	.12	.056	15.6	.11	.057	15.0	.10	.054	13.9
E	.46	.028	60.6	.46	.028	60.9	.46	.028	61.8
Total	.76	.023	100	.76	.023	100	.74	.022	100

var: absolute variance; se: standard error of variance; %: variance per component in per cent of total variance; N=3,168. Coefficient for assortative mating is .5195; if models did not converge with this factor slightly higher coefficients were used.
